# Vowel generation for children with cerebral palsy using myocontrol of a speech synthesizer

**DOI:** 10.3389/fnhum.2014.01077

**Published:** 2015-01-22

**Authors:** Chuanxin M. Niu, Kangwoo Lee, John F. Houde, Terence D. Sanger

**Affiliations:** ^1^Department of Rehabilitation, School of Medicine, Ruijin Hospital, Shanghai Jiao Tong UniversityShanghai, China; ^2^Department of Biomedical Engineering, University of Southern CaliforniaLos Angeles, CA, USA; ^3^Department of Otolaryngology - Head and Neck Surgery, University of California, San FranciscoSan Francisco, CA, USA; ^4^Biokinesiology, University of Southern CaliforniaLos Angeles, CA, USA; ^5^Neurology, University of Southern CaliforniaLos Angeles, CA, USA

**Keywords:** electromyography (EMG), speech impairment, speech synthesis, Cerebral Palsy, non-linear Bayesian filtering

## Abstract

For children with severe cerebral palsy (CP), social and emotional interactions can be significantly limited due to impaired speech motor function. However, if it is possible to extract continuous voluntary control signals from the electromyograph (EMG) of limb muscles, then EMG may be used to drive the synthesis of intelligible speech with controllable speed, intonation and articulation. We report an important first step: the feasibility of controlling a vowel synthesizer using non-speech muscles. A classic formant-based speech synthesizer is adapted to allow the lowest two formants to be controlled by surface EMG from skeletal muscles. EMG signals are filtered using a non-linear Bayesian filtering algorithm that provides the high bandwidth and accuracy required for speech tasks. The frequencies of the first two formants determine points in a 2D plane, and vowels are targets on this plane. We focus on testing the overall feasibility of producing intelligible English vowels with myocontrol using two straightforward EMG-formant mappings. More mappings can be tested in the future to optimize the intelligibility. Vowel generation was tested on 10 healthy adults and 4 patients with dyskinetic CP. Five English vowels were generated by subjects in pseudo-random order, after only 10 min of device familiarization. The fraction of vowels correctly identified by 4 naive listeners exceeded 80% for the vowels generated by healthy adults and 57% for vowels generated by patients with CP. Our goal is a continuous “virtual voice” with personalized intonation and articulation that will restore not only the intellectual content but also the social and emotional content of speech for children and adults with severe movement disorders.

## Introduction

Children with brain injury in the perinatal period, usually referred as Cerebral Palsy (CP), are often left with a combination of weakness, spasticity, dystonia, dyspraxia, and other motor disorders (Cans, 2000). These disorders in CP result from dysgenesis or injury to developing motor pathways in many components of central nervous system, including the cortex, basal ganglia, thalamus, cerebellum, brainstem, central white matter, or spinal cord. Most patients with CP struggle to maintain limb postures or perform voluntary movements due to increased muscle tone or weakness. Concomitant issues in emotional and behavior are also common in CP (Bax et al., 2005). In the most severe cases, the motor disorders in CP can prevent all meaningful voluntary movements of the patient (Sanger et al., 2003), and more than 80% of children with dyskinetic or tetraplegic CP suffer from speech impairments (Odding et al., 2006). While new therapies such as stem cells hold great promise for the treatment of early brain injuries, full restoration of speech for children with CP remains unlikely.

We use the term “language” as the content of human communication, either spoken or written, consisting of the use of words in a structured and conventional way (Oxford English Dictionary); while the term “speech” as the motor function required for vocalizing human language. It is common that patients with CP may have normal language ability (via writing or assistive devices) but are completely incapable of producing speech. Our ultimate goal is to allow children with CP to create intelligible English speech using a portable synthesizer controlled in real-time by body signals such as muscle activity. For children with CP who have preserved language skills but impairment of control of vocal tract musculature, our aim is to create a “virtual voice” to enable them to express language using other muscles for which they may have better voluntary control. The primary engineering challenges for restoring speech are threefold: (1) extracting controllable signals from a diseased neurological system, (2) using these signals to rapidly synthesize sounds resembling human speech, and (3) providing personalized ways of speaking. The third challenge is particularly important for children, since language is used by children for social interaction and emotional communication, much more than for declarative statements (Wing and Gould, 1979; Van Lancker et al., 1989; Patel and Schroeder, 2007). It has been shown that muscle patterns in children with CP are distorted by co-contraction (Young et al., 2011a,b), signal-dependent noise (Sanger et al., 2005; Sanger, 2006), and weakness, which reduce the speed and accuracy of control and lead to a limited effective bandwidth of the voluntary signal (Sanger and Henderson, 2007). Therefore, the challenge is to allow sufficient flexibility in the voice output despite limited bandwidth of the voluntary control signals. We thus seek to maximize the controllability of the produced speech, while minimizing the need for precise control of muscles.

Myocontrol, the control of prosthetic devices using surface electromyographic (EMG) signals, holds great promise for speech production. Previous studies provide the essential support that EMG from limb muscles provides an excellent signal that children with CP can often control (van der Heide et al., 2004). The flexibility and accuracy of muscle activity could potentially approach the quality and flexibility required in speech control. Furthermore, signal processing could potentially transform abnormal muscle patterns of children with CP into much more precisely controllable signals with significantly better performance. In speech science, EMG has been adopted in various studies including the real-time recognition of impaired speech (Jorgensen et al., 2003; Jou et al., 2007); EMGs from neck strap muscles have also been successfully used for driving an artificial larynx in patients who receive laryngectomy (Stepp et al., 2009). In these applications, however, patients have normal neural control and need only bypass abnormal muscles or biomechanics. But for children with CP and absent speech, the neural control itself is impaired so that EMG from the neck muscles is not expected to function for control of speech. Compared to muscles around the neck and vocal tract, limb muscles are defined more clearly and therefore easier to attach surface EMG recording electrodes. Here we will leverage the fact that significant amount of voluntary control can still be reconstructed from limb muscles of children with CP.

Successful application of myocontrol has been limited by the variability in raw EMG signals and the consequent poor quality of estimates. Most existing methods for EMG processing stem from the idea that EMG can be treated as an amplitude-modulated signal with band-limited noise (Hogan and Mann, 1980a,b). Based on this perspective, a procedure of high-pass, rectify and low-pass filtering has been developed and widely adopted for EMG processing (Evans et al., 1984; Merletti, 1999). Using this procedure, however, it is difficult to obtain online control signals that are both responsive and smooth, which is extremely critical for real-time applications such as restoring movement functions. With our recently developed techniques of non-linear Bayesian filtering (Sanger, 2007), we are able to extract high-bandwidth, low-latency control signals from raw surface EMG. The technique has been applied to studies of biofeedback (Bloom et al., 2010) and motor control (Young et al., 2011b) for children with dyskinetic CP. It provides another essential support for using myocontrol in speech production for CP. See Materials and Methods for details.

In addition to the problem of control, speech restoration for children with CP also requires a technology that can synthesize speech-like voices with a small number of control parameters, and yet still allow for flexible voice output. The technology of speech synthesis has been of interest for more than 200 years (von Kempelen, 1791), and it has evolved into three categories of synthesis approaches: concatenative, articulatory and formant-based. The simplest way to produce synthetic speech is to play back pre-recorded pieces of natural speech following pre-determined concatenations. This *concatenative* approach produces very high quality of voice in text-to-speech applications (Taylor et al., 1998), but pre-recorded voices are usually unavailable from children with CP. Even though in some cases the patients' voice can be pre-recorded, such systems usually require accurate selection of speech elements; thus the control task may be harder than necessary.

As a continuous alternative to concatenative synthesis, *formant-based* synthesis uses relatively few control parameters and allows for full control of intonation and inflection (Klatt, 1980). In the case of vowels, for instance, it has been suggested that frequencies of the lowest two formants (F1 and F2) are sufficient for vowel intelligibility, while formant bandwidths and other parameters are less important (Stevens, 2000; Ladefoged and Johnstone, 2010). Therefore, in the case of vowel synthesis, it becomes possible to reduce the number of parameters for control in CP using a low dimensionality of control inputs. In a recent study (Larson et al., 2013), the investigators succeeded in using two channels of surface EMG from orbicularis oris muscles to control F1 and F2 for vowel synthesis. The EMGs were categorized into pre-defined syllables and therefore would not provide continuous auditory feedback. In this study, because we convert EMGs moment-by-moment to continuously varying formant frequencies, our system will produce continuous voice even though the target phoneme has not yet fully developed. As a result, even though real-time myocontrol allows significant flexibility that could potentially lead to personalized intonation and articulation, the very first step should be testing the intelligibility of vowels using myocontrol.

Ultimately, the goal of myocontrolled speech synthesis is to generate both vowels and consonants in continuous speech. In reaching this goal, generation of consonants presents several important challenges. First, many consonants (e.g., plosives and fricatives) are rapid, dynamically changing acoustic events. Such consonants thus require high bandwidth in the control signal to produce the fast temporal transitions. Second, many consonants involve controlling more acoustic features than just F1 and F2, e.g., frication noise for fricatives and F3 for /r/ (Espy Wilson, 1992; Heinz and Stevens, 2005). Our technique of myocontrolled vowel synthesis can be directly used for producing liquid consonants (or semi-vowels) due to the acoustic similarity between vowels and liquid consonants (Ladefoged and Johnstone, 2010). For other consonants that have dynamics and richer acoustic features (plosives, fricatives, etc.), we expect that certain dimensionality reduction methods will be required to simplify control of the formant trajectory and sound production process such that the consonant production can be mapped to activation of a limited number of muscles. The focus of the current study, however, is to answer the initial feasibility question of whether vowel synthesis can be controlled in real-time using non-speech muscles.

This paper first introduces the design and methodology of real-time vowel generation using EMG from non-speech muscles. Next, we describe testing the quality of myocontrolled speech synthesis in 10 healthy adults and 4 patients with dyskinetic CP. The two populations are not age matched since our purpose is validation of myocontrolled vowel-synthesis in a wide range of subjects. We hypothesize that for healthy adults the vowels synthesized via myocontrol will be recognized by naïve listeners with statistical significance; in patients with CP, the intelligibility is expected to be lower than healthy adults, but the listeners should still be able to identify the vowels with statistical significance. The fraction of vowels correctly identified by 4 naive listeners is calculated to test the feasibility of intelligible speech restoration using myocontrol. Preliminary results have been reported as an abstract (Niu et al., 2013). Our main innovations are (1) using non-linear Bayesian filtering to extract high-bandwidth, low variability control signals and (2) mapping the vowel generation to moving a cursor on a 2D plane, which is intuitive even for children with CP.

## Materials and methods

All research participants signed written informed consent to participate as well as U.S. Health Information Portability and Accountability Act (HIPAA) authorization for use of medical and research records, according to approval of University of Southern California Human Subjects Review Board. Both healthy subjects and patients with CP were recruited. Healthy subjects without known neurological disease were recruited as control. Patients with CP were required to have normal cognition such that they could understand the experimental instructions, and at least one side of their upper extremities should show motor deficits. Ten healthy adult subjects (7 male, 3 female, age 21–29 years), four subjects with dyskinetic CP (2 male, 2 female, age 12–20 years) and four naïve listeners (2 male, 2 female, age 25–30 years) were recruited. The clinical diagnosis and motor deficit analyses of 4 patients with CP are summarized in Table 1.

**Table 1:** **Diagnosis of four patients with dyskinetic CP**.

**Patient**	**Gender**	**Age**	**Clinical diagnosis**	**Motor deficits**
1	Male	12	Ataxia, severe dysarthria, mild bradykinesia, normal cognition	Slow movements, hand dystonia, intention tremor, bilateral hand athetosis; Mild gait instability
2	Male	18	Left arm dystonia, dyspraxia, normal cognition	Stiff arms and hands, impaired fine movements due to dyspraxia; Tight hamstring during walking, excessive dorsiflexion in ankles, hips adducted in stance
3	Female	20	Generalized secondary dystonia, normal cognition	Occasional shoulder jerk, mild torticollis, writing with stiff posture and easily fatigued; Normal walking
4	Female	12	Right hemiplegia, chorea, right eye impaired vision, mild to moderate cognition	Tremor of right upper extremity, very stiff right arm and hand; Impaired gait with foot drop

### Synthesizing vowel in real-time by controlling formants

**Figure 1:**
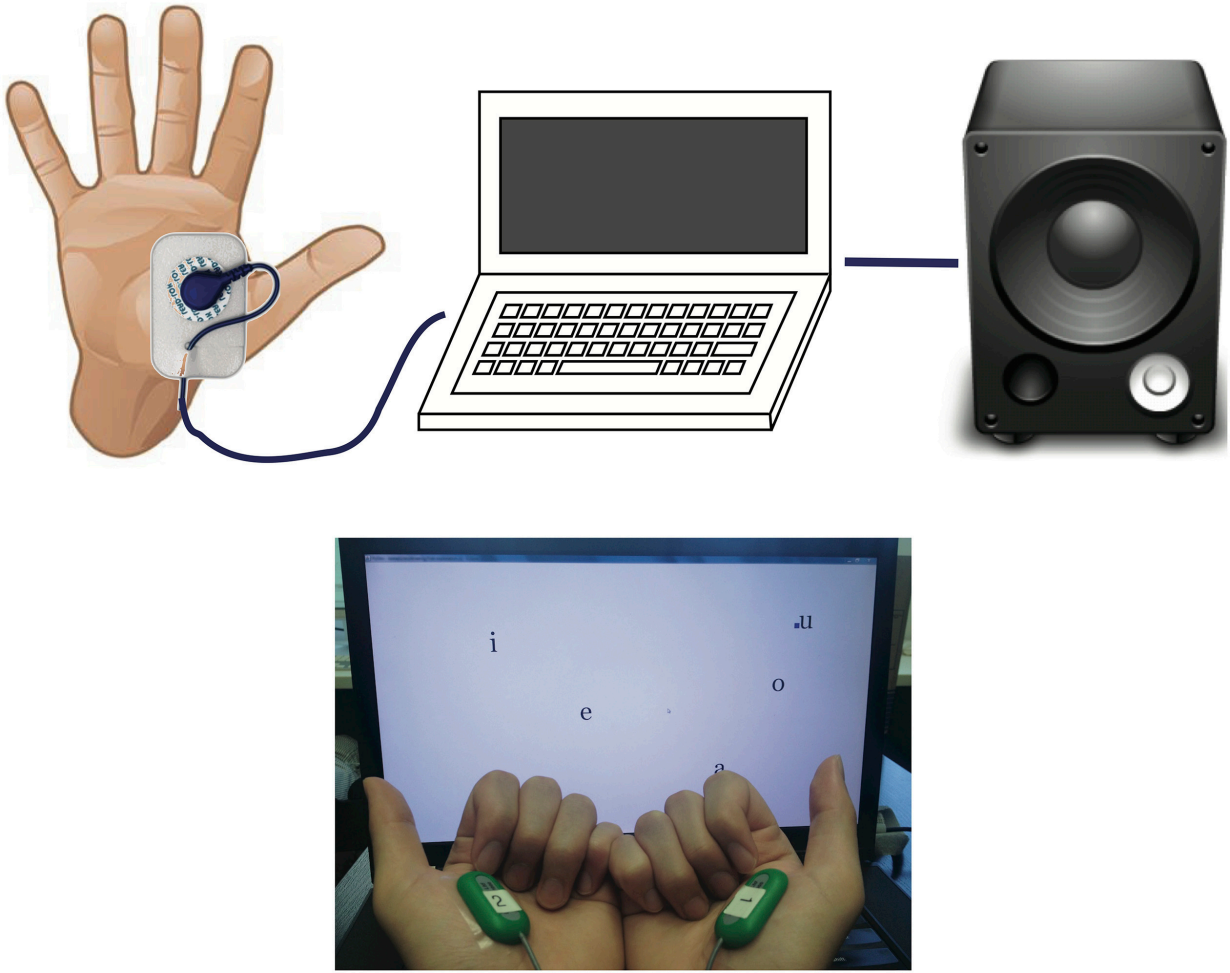
**Design of the system (top) and a snapshot of the working set-up (bottom)**. The EMG electrodes are placed on the flexor pollicis brevis muscles of both hands. Pinching the thumb and index finger will activate the EMG to move both the screen cursor and formant frequency.

The overall design of our myocontrolled formant-based synthesis platform is shown in Figure 1. Raw muscle EMGs are monitored using surface EMG electrodes (Biometrics SX230) attached over the belly of the chosen muscle. EMG signals are sampled at 1 kHz and processed online using a non-linear Bayesian filter. The screen provides visual feedback of the muscle activity, and also shows the level of activity required for a given vowel. Both auditory and visual feedback are continuously provided during experimental tests.

In human speech, vowel production can be described as a broadband sound source generated at the glottis (i.e., the glottal source) that is filtered by the vocal tract (e.g., the pharynx, tongue, palate, teeth, lips) (Titze, 2000). The glottal source is periodic with a fundamental frequency F0 that is lower for male voices and higher for female voices. The vocal tract has several resonances that speakers change by moving the articulators, especially the tongue. These resonances filter the glottal source, so that the output speech spectrum has harmonics of the pitch modulated by broad peaks of the vocal tract resonances called formants. The frequencies of the lowest two formants (F1 and F2) determine which vowel is being spoken, while higher formants usually reflect non-phonetic speaker characteristics (e.g., vocal tract length) (Stevens, 2000; Ladefoged and Johnstone, 2010). Thus, in formant-based speech synthesis, we can artificially synthesize different vowels by tuning the lowest two resonances of a filter driven by a broadband periodic (e.g., impulse trains) acoustic source (Parsons, 1987; Stevens, 2000).

In this project, we adapted an open-source formant-based synthesizer (Wavesurfer, KTH Royal Institute of Technology, Sweden) such that F1 and F2 can be directly controlled over an ethernet link in real-time. The reason of selecting a well-developed synthesizer is to keep our goal modest, such that if the feasibility test fails it is likely due to our myocontrol design but not the synthesizer. The source code of the Wavesurfer synthesizer is located at http://www.speech.kth.se/wavesurfer/formant/. The adapted version is available upon request from the authors of this paper.

### Non-linear bayesian filtering of EMG

Under the assumption that the rectified EMG signal results from random depolarization events of multiple muscle fibers, the average amplitude of rectified EMG in a small time window will be proportional to the number of depolarization events during that time. One representative non-linear model of such depolarization events is a non-homogeneous Poisson process with n events per second, controlled by the muscle drive *x*:

(1) $$P\left(EMG|x\right)\propto \frac{x^{n}e^{-x}}{n!}$$

where the driving signal x is unknown and thus must be estimated by the filtering algorithm.

Since the drive signal x is determined by voluntary behavior, we model this behavior as a jump-diffusion process that includes the possibility of gradual changes in muscle drive with occasional sudden jumps at the time of force onset or offset:

(2) $$dx=\alpha \left(dW\right)+\left(U-x\right)dN_{\beta }$$

where the stochastic differential equation is to be interpreted in the Ito sense, *dW* is the differential of a standard Brownian motion, *dN*_β_ is the differential of a counting process with rate β events per second, and x is a random variable uniformly distributed on [0,1]. Equation 2 models the gradual drift of x determined by the drift rate α, and rare jumps that occur at transitions in the counting process *dN*_β_, at which times the value of *x* will change to a new random value drawn from the distribution of *x*.

Using Equation 1, we can derive a posterior estimate for *x* based on measurement of EMG and a prior estimate of the density of *x* using Bayes' rule. Between measurements of EMG, *x* will change according to the stochastic differential equation Equation 2, and the distribution of *x* will propagate forward in time according to a corresponding partial differential equation similar to the Fokker-Planck equation. After each measurement, the maximum a posteriori estimate of *x* is calculated using Bayes' rule, and this provides the output estimate from the algorithm at that time.

**Figure 2:**
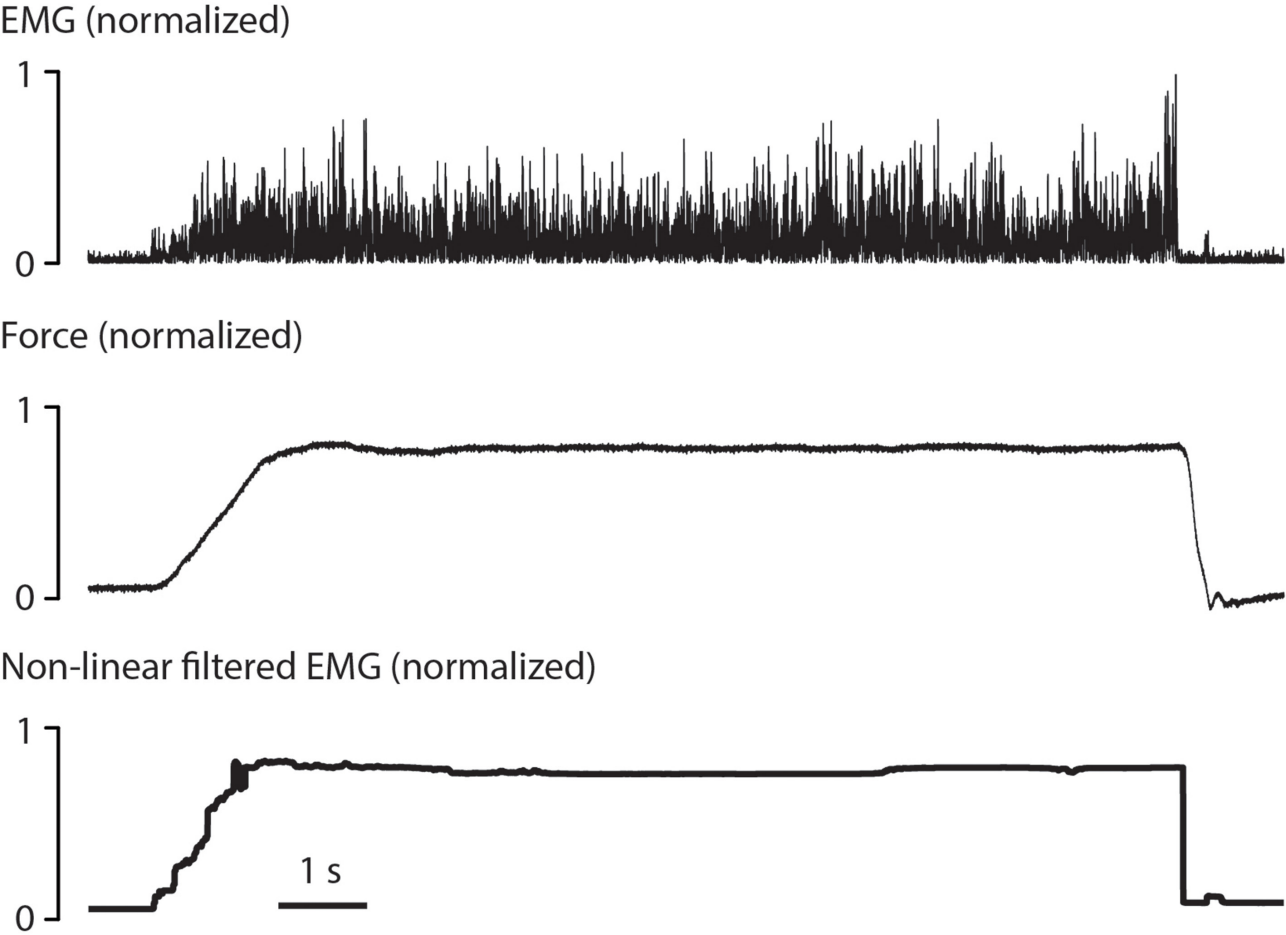
**Example of non-linear Bayesian filtering, re-analyzed and produced from previous dataset (Sanger, 2007)**. A rectified surface EMG signal **(top)** was recorded during 10 s of isotonic contraction in the biceps. The corresponding force **(middle)** was both fast-changing and smooth. The signal produced by non-linear Bayesian filter **(bottom)** showed comparable rising time and falling time, and maintained small variability during the contraction.

An example of non-linear Bayesian filtering applied to rectified EMG is shown in Figure 2. As can be seen, the output from non-linear Bayesian filter has captured the rapid changes in force, but still with sufficient smoothness and dynamic range. For details about this EMG model and filtering algorithm refer to Sanger (2007). There exist a variety of EMG filtering algorithms that may all allow myocontrolled speech synthesis. We adopt non-linear Bayesian filtering due to its benefits of fast time-domain response and low variability. Detailed comparison between EMG filtering algorithms for speech synthesis may be tested if the basic feasibility is proven.

### Formant placement is a 2D reaching movement

**Figure 3:**
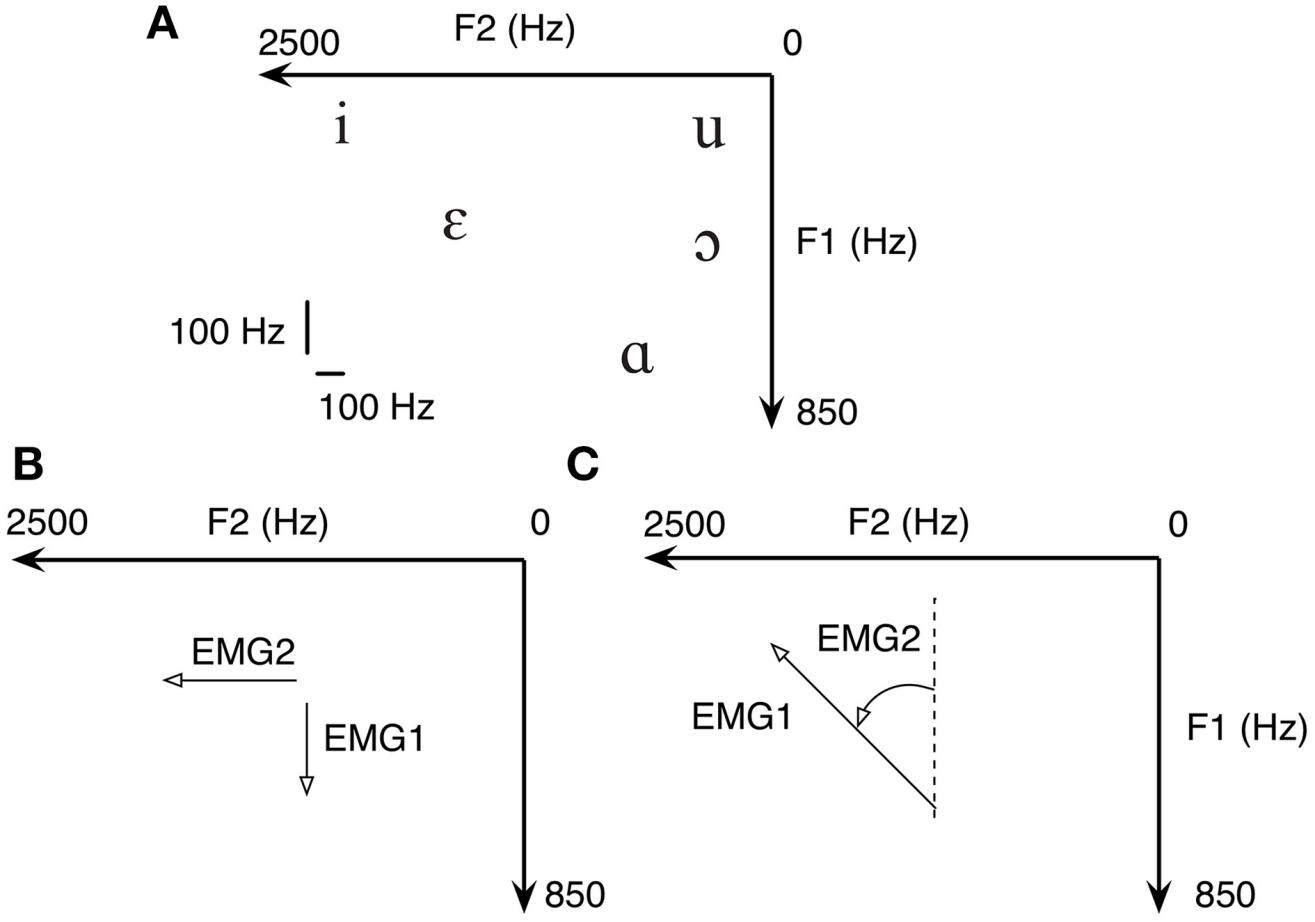
**(A)** In formant-based speech synthesis, each vowel represents a point on the 2D plane defined by F1 and F2. Therefore, five common U.S. English vowels are one-on-one mapped to five targets on a 2D plane. Both the Cartesian **(B)** and polar **(C)** transform from EMG to formant are tested due to the non-negativity of EMG signals.

The five common English vowels and the frequencies of their lowest two formants (F1 and F2) are shown in Table 2 according to Hillenbrand et al. (1995) On a plane defined by F1 and F2, each of the five vowels represents a point, as can be seen in Figure 3A. The mapping between vowel and locations on a 2D plane is visualized and shown to the subject for learning the use of myocontrolled vowel production. It is important to realize that perfect accuracy is not required; vowels will be recognizable in a region near the targets, and the tolerance for error will be greater when vowels are incorporated as part of a word or phrase in which meaning can be inferred.

**Table 2:** **Average formant frequencies (Hz) for U.S. English vowels produced by 45 males (from Hillenbrand et al., 1995), with the corresponding EMG magnitude under Cartesian and polar transform**.

**Vowel IPA**	**Example**	**F1**	**F2**	**EMG1 Cart.**	**EMG2 Cart.**	**EMG1 polar**	**EMG2 polar**
/ɑ/	“hot”	768	1333	0.71^*^	0.30	0.28	0.62^*^
/e/	“get”	580	1799	0.44	0.52	0.23	0.27
/i/	“bee”	342	2322	0.10	0.77^*^	0.72^*^	0.21
/ɔ/	“bought”	652	997	0.54	0.14	0.51	0.72^*^
/u/	“boot”	378	997	0.15	0.14	0.53	0.80^*^

* *High muscle contraction level required*.

To familiarize with the task without the need to learn myocontrol simultaneously, we first asked all subjects to repetitively move between /i/ and /ɑ/ using the finger to swipe across the surface of a touch-screen. To demonstrate the feasibility of placing F1 and F2 using myocontrol, we asked the same subject to move the cursor on the formant plane using flexor pollicis brevis EMGs from both hands. The EMG signals were transformed to F1 and F2 using the Cartesian transform (explained below). See Results for details.

### Control strategies for placing formants

Since F1 and F2 frequencies represent points on a 2D surface, filtered EMGs must be mapped to positions within the same surface. Due to the non-negativity of filtered EMG, we chose two straightforward position mappings either in Cartesian coordinates within the first quadrant, or polar coordinates within the entire 2D surface. Cartesian transform is given by:

(3) $$F_{1}=\left(F_{1}^{hi}-F_{1}^{lo}\right)EMG_{1}+F_{1}^{lo}$$

(4) $$F_{2}=\left(F_{2}^{hi}-F_{2}^{lo}\right)EMG_{2}+F_{2}^{lo}$$

where *F*^*hi*^_1_, *F*^*lo*^_1_, *F*^*hi*^_2_ and *F*^*lo*^_2_ are the boundary frequencies chosen such that all vowels listed in Table 2 are reachable with normalized *EMG*_1_, EMG_2_ ϵ [0,1]. When using the Cartesian transform, a dot cursor representing the current coordinate of (*F*_1_, *F*_2_) was shown on the screen (Figure 3B).

Another way of transforming non-negative normalized EMG into formant space is to use polar coordinates. The transform is given by:

(5) $$F_{1}=\frac{F_{1}^{hi}+F_{1}^{lo}}{2}+K_{F1}EMG_{1}sin\theta_{E}$$

(6) $$F_{2}=\frac{F_{2}^{hi}+F_{2}^{lo}}{2}+K_{F2}EMG_{1}cos\theta_{E}$$

(7) $$\theta_{E}=2\pi EMG_{2}+\frac{\pi }{2}$$

where *K_F1_* and *K_F2_* are scaling factors for adjusting the size of the ellipse defined by the transform. When using the polar transform, the visual feedback was provided by a vector line, of which the magnitude was primarily driven by *EMG*_1_ while the angle driven by *EMG*_2_ (Figure 3C). In the current setup we choose that when *EMG*_1_ = 1.0 and *EMG*_2_ = 0.0 the vector is pointing to the negative direction of *F*_1_.

The normalized EMGs required for each vowel are listed in Table 2, calculated according to Equations (3–7). When using Cartesian transform, the cursor starts from the top-right corner of the screen; when using polar transform, the cursor starts from the center of the screen. It is worth noting that the origin of the coordinate system directly affects the magnitude of *EMG*_1_ and *EMG*_2_ required for each vowel. Therefore, reaching certain targets may require an EMG level higher than 60% of maximum voluntary contraction (MVC, see asterisk terms in Table 2), which has been known to cause fatigue (Bigland-Ritchie et al., 1981). Simple increasing the gain of non-linear filter will reduce the maximum EMG level, but this will reduce the accuracy when producing low level EMGs. Therefore, one goal in the future is to minimize the EMG amplitude requirement while maintaining the accuracy at low EMG levels.

### Experimental procedure of randomized vowel generation

Subjects were seated in front of a laptop computer, with both hands resting on the knees. Activities from bilateral upper-limb muscles were monitored using two surface EMG electrodes, one for each hand. For healthy subjects with normal hand and arm function, signals from brachioradialis (BR, an elbow flexor) and flexor pollicis brevis (FPB, a thumb flexor) muscles were separately tested for myocontrol; for subjects with dyskinetic CP who had trouble using hand muscles, only BR was tested. In FPB conditions, subjects were instructed to pinch their thumb and index finger to activate the muscle; in BR conditions, the muscles were activated by the subjects lifting their forearms against the desk. Before the experiment, raw EMG amplitude was normalized to MVC (maximum voluntary contraction) determined for each electrode using the highest activation in any 250 ms period during five 5-s attempts, with visual feedback and encouragement.

**Figure 4:**
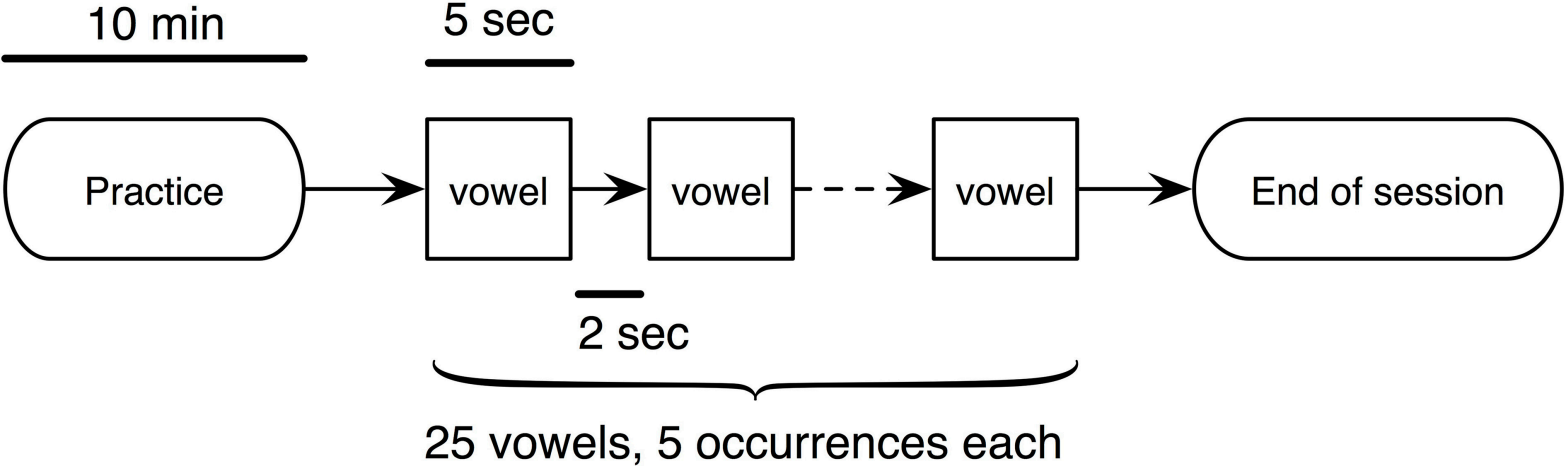
**The experimental procedure for testing the quality of vowel synthesis**. Two sessions (Cartesian or polar) were given to each subject. Both the healthy subjects and patients received the 10-min practice for familiarizing with the device.

All subjects received two test sessions (Cartesian transform and polar transform) in random sequence, illustrated in Figure 4. During each session, the subject was first given a 10-min practice to familiarize with vowel generation. During practice, the locations in formant space of all 5 vowels were displayed on the screen, and continuous audio feedback was provided. Subsequently, subjects were required to produce a series of 25 movements to targets in formant space corresponding to random vowels containing 5 occurrences of each common English vowel (/ɑ/, /e/, /i/, /ɔ/, /u/). In each trial, the target in formant space was shown, and the subject was allowed 5 s to produce the target vowel. A 2-s break was given between each trial.

The 5-s sound clips recorded from each trial were played to four naive listeners (native U.S. English speakers) to identify the attempted vowel. At the beginning of each clip, the initial sound represent the two formants when *EMG*_1_ = 0, *EMG*_2_ = 0; the initial sound then transitioned to the steady-state sound produced by each subject. The listener was informed that during the 5 s the subject was trying to speak one vowel out of five possibilities. The listener was forced to make a choice by inferring from the steady-state sound and ignoring the transition. The fraction of vowels correctly identified by the listener, given by *N_correct_*/*N_total_*, was calculated to show the overall quality of myocontrolled vowel generation.

### Statistical analysis

The responses from each of the 4 naive listeners are analyzed using Cohen's κ-test (Viera and Garrett, 2005) to determine whether the synthesized vowels are intelligible. The purpose of Cohen's κ-test is to identify whether there is a difference in responses between the naive listener and an imaginary perfect listener who has exact knowledge of which vowel was spoken. In our case the listener was required to pick an answer from 5 candidate vowels, therefore an imaginary perfect listener would achieve 100% fraction of correctness, while a random guesser would be expected to achieve, on average, 20% correctness. In order to test the agreement among 4 listeners, we also analyzed their responses using Fleiss's κ-test (Fleiss, 1971).

## Results

**Figure 5:**
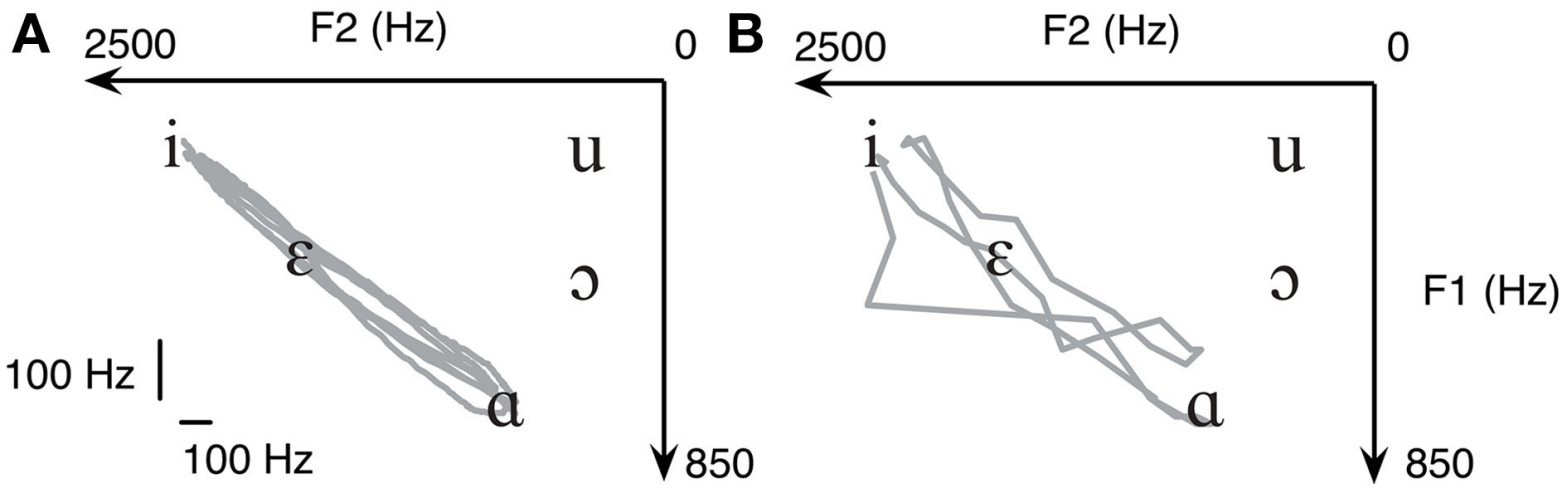
**When making repetitive reaching between /i/ and /ɑ/ on a touchscreen, the trajectory (A) automatically produces the sound of “ee-ah-ee-ah.”** The same task can be done using myocontrol **(B)**, where the trajectory has significantly higher variability but still produces “ee-ah-ee-ah.” Data from a healthy subject are shown.

We first asked the subjects to repetitively move between /i/ and /ɑ/ using the finger to swipe across the surface of a touch-screen. The trajectories resulting from the finger movements of a healthy subject are displayed in Figure 5A. In agreement with previous studies (Atkeson and Hollerbach, 1985; Uno et al., 1989), the trajectories of finger swiping movements were clustered around a straight line, even though the subject was free to choose any possible trajectory. Once the trajectory shown in Figure 5A was connected to our speech synthesizer it immediately produced the vowel sequence of “ee-ah-ee-ah.” It is hence suggested that when using formant-based synthesis, producing a target vowel can be equivalently interpreted as reaching to a target position within a 2D plane.

We then asked the same subject to move the cursor on the formant plane using flexor pollicis brevis EMGs from both hands using the Cartesian transform. The myocontrolled trajectory is shown in Figure 5B. As can be seen, although the variability of the trajectory is significantly higher, the two targets (/i/ and /ɑ/) were still reached. This demonstrates the feasibility of using EMGs to move a cursor on the 2D formant plane. Notice the abrupt jump in the bottom-left part of the trajectory shown in Figure 5B, this is because non-linear Bayesian filtering allows for rapid jumps even though the main purpose is still acquisition of smooth control signals. We argue that the task remains intuitive to the subject, which will facilitate learning.

**Figure 6:**
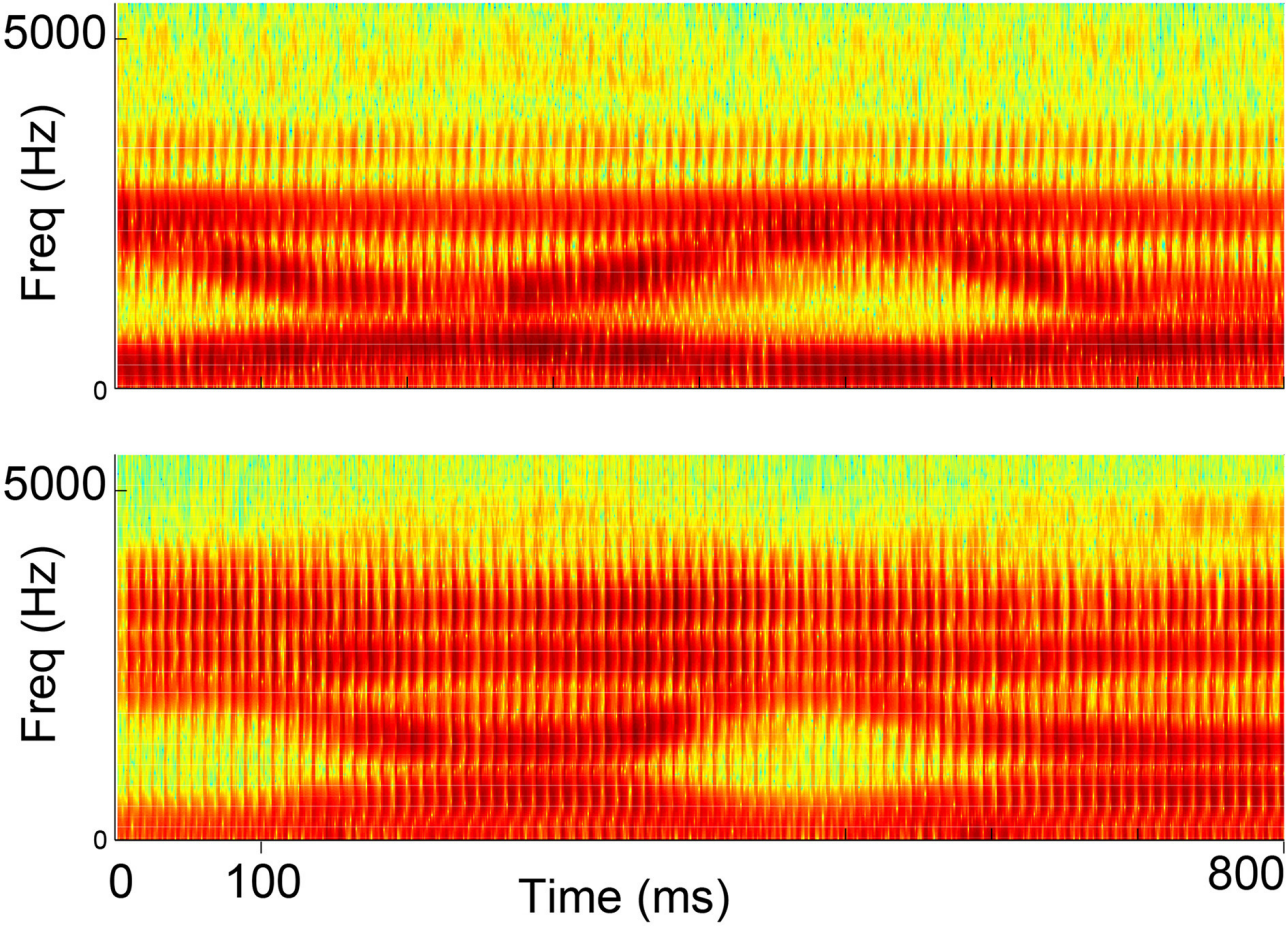
**Spectrograms of synthesized speech generated from myocontrol (top) and natural human speech (bottom) for the vowel sequence “ee-ah-ee-ah”**.

We first compared the spectrogram of a vowel sequence “ee-ah-ee-ah” produced by both myocontrolled synthesizer and natural human speech of a healthy subject (Figure 6). It can be seen that the spacing between the lowest two formants are qualitatively similar. This is not surprising since the synthesizer is designed to closely match human voice, but the test is still useful for the validation of whether subjects can indeed drive the synthesizer using limb muscles.

The responses from the naive listener for all 10 healthy subjects are summarized in Table 3. For each condition, the fraction of correctness is averaged across all vowels produced by all 10 subjects. As can be seen, in all cases the fractions of success are higher than 60%, suggesting that it is feasible to produce intelligible English vowels using myocontrol. Cohen' κ-tests show that in all cases the κ-values are higher than 0.50, suggesting at least “Moderate” (“Almost Perfect” when using Cartesian transform) agreement between the naïve listeners and an imaginary perfect listener.

**Table 3:** **The responses of 4 listeners to vowels from 10 healthy subjects and 4 patients with dyskinetic CP**.

**Group**	**Muscle**	**Transform**	**Correct (%)**	**κ(*SD*)**	Quality^*^
Healthy	BR	Cart.	88.02	0.853(0.042)	Almost perfect
		polar	61.40	0.520(0.014)	Moderate
	FPB	Cart.	89.20	0.865(0.021)	Almost perfect
		polar	71.20	0.641(0.038)	Substantial
CP	BR	Cart.	57.00	0.473(0.053)	Moderate
		polar	46.75	0.281(0.050)	Fair

* *The quality of raters agreement is assessed according to Landis and Koch (1977)*.

**Figure 7:**
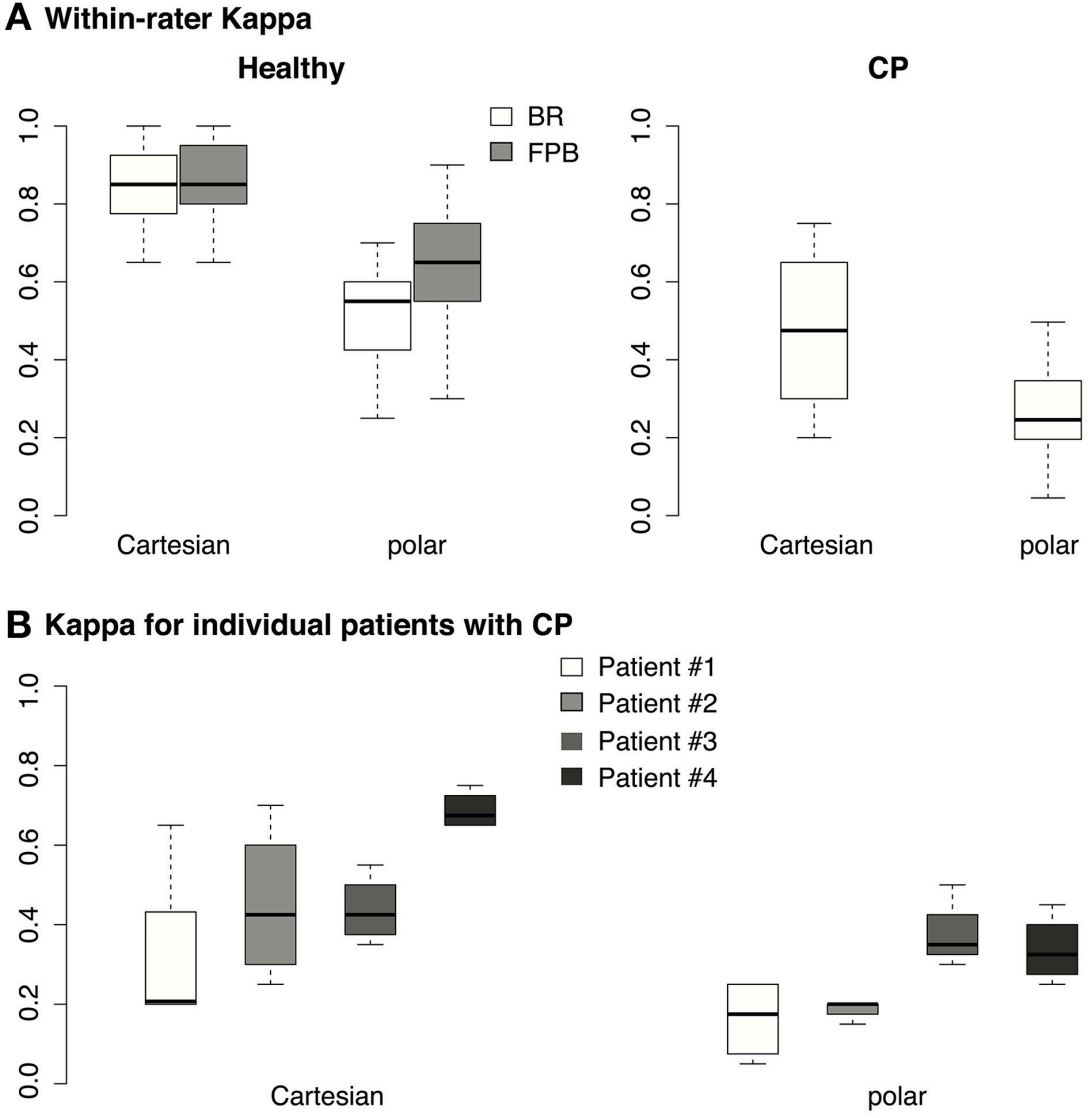
**(A)** Cohen's κ calculated from the responses of 4 listeners for the 10 healthy subjects. Each data point represents the responses from 1 listener across 25 vowels. For healthy subjects, each box covers 4 (listener) × 10 (subject) = 40 data points. **(B)** Cohen's κ calculated from the responses of 4 listeners for 4 patients with CP, each box covers 4 (listener) × 4 (patient) = 16 data points.

Two-Way repeated measures ANOVA shows that in healthy subjects (Figure 7A, healthy), Cohen's κ is significantly higher when using Cartesian transform compared with polar transform [main effect of transform, *F*_(1, 9)_ = 169.7, *p* < 0.00001]; also our design favors the finger flexor (FPB) over the elbow flexor (BR) [main effect of muscle, *F*_(1, 9)_ = 9.831, *p* < 0.05]. The interaction between transform and muscle is also significant [*F*_(1, 9)_ = 20.51, *p* < 0.01]. These results suggest that both the EMG-formant mapping and muscle selection are important for future improvements of myocontrolled speech synthesis.

For the patients with dyskinetic CP, the responses of the listeners are also shown in Table 3. Since the subjects with dystonia had difficulty controlling their FPBs, only brachioradialis muscles (BR) were tested. Although the fraction correct for subjects with dyskinetic CP was lower than for healthy subjects, the naive listeners were still able to identify almost half of the vowels generated by subjects with dyskinetic CP. Cohen' κ-test also shows either “moderate” or “fair” agreement between the naive listeners and an imaginary perfect listener, suggesting that the vowels were unlikely to have been identified from pure speculation. Similar to healthy subjects, One-Way repeated measures ANOVA shows that for these 4 patients (Figure 7A, CP), our design favors Cartesian transform compared to polar transform [main effect of transform, *F*_(1, 3)_ = 10.9, *p* < 0.05].

The performances of individual patients measured in κ are shown in Figure 7B. Although patient #4 performed better than other patients, the κ-values of all patients across all listeners using Cartesian transform were higher than 0.3 [one-tailed *t*-test, *t*_(15)_ = 3.5, *p* < 0.01], suggesting a Fair quality of fit according to Landis and Koch (1977). Similarly, the κ-values for patients using polar transform were higher than 0.2 with Fair quality of fit [one-tailed *t*-test, *t*_(15)_ = 2.2, *p* < 0.05]. It is unlikely that the statistical outcomes were due to the outperforming outliers.

Since both visual and auditory feedback were provided during the task, we compared the performance measured by both visual error and auditory intelligibility. We measured the visual error (*VE*) as the distance between the instantaneous cursor position and the final target in the 2D formant space:

(8) $$VE(t)=\sqrt{{\left(F_{1}\left(t\right)-F_{10}\right)}^{2}+{\left(F_{2}(t)-F_{20}\right)}^{2}}$$

**Figure 8:**
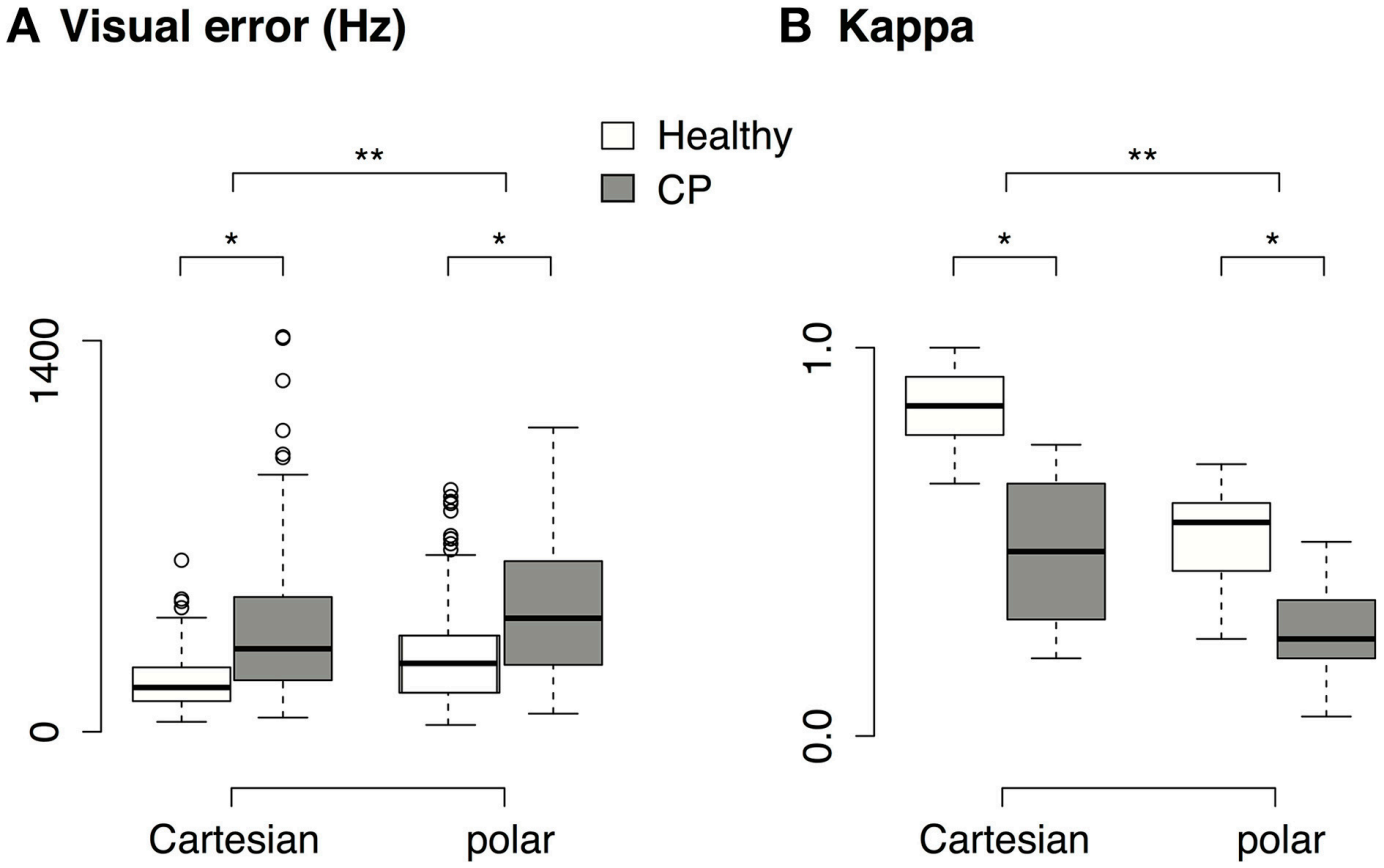
**(A)** Visual error across population and transform. Only data from the brachioradialis muscle are shown, since the patients with CP were only tested with this muscle. **(B)** Cohen's κ across population and transform. Only data from brachioradialis muscle are shown. ^*^p < 0.05, ^**^p < 0.001.

The average *VE* during the last second of the 5-s task is shown in Figure 8A. Two-Way repeated measures ANOVA shows that the visual error was significantly higher in patients than in healthy subjects [main effect of population, *F*_(1, 3)_ = 10.9, *p* < 0.05]. Suppose a linear measurement of the performance (p) that is inversely proportional to the visual error (*VE*):

(9) $$p=\frac{1}{VE}$$

The relative increase in *VE* is related to the relative decrease in *p*:

(10) $$\Delta p=\frac{p_{2}-p_{1}}{p_{1}}=\frac{\Delta VE}{1+\Delta VE}$$

(11) $$\Delta VE=\frac{VE_{2}-VE_{1}}{VE_{1}}$$

Take Cartesian transform as an example, the mean visual error increased by 114.9% from healthy subjects (179.42 ± 94.26, mean ± sd) to patients with CP (385.65 ± 293.93, mean ± sd), therefore the measurement of performance should decrease by 53.3%. Nevertheless, the performance (measured in κ, see Figure 8B) in myocontrolled vowel generation only decreased by 44.7% from healthy subjects (0.85 ± 0.1, mean ± sd) to patients with CP (0.47 ± 0.2, mean ± sd). Similar patterns were found in polar transform. This suggests that our paradigm could tolerate a certain amount of variability in the subject's input. It is also suggested that the subject did not try to minimize the visual error during the task, but instead they tried to rely on the auditory feedback and use the auditory intelligibility of the vowel as the control criteria.

Inter-rater agreements measured in Fleiss' κ are shown in Table 4. For healthy subjects the agreement level represented by κ-value is higher than 0.80 when using Cartesian transform, suggesting “Almost Perfect” agreement among listeners according to Landis and Koch (1977); when using polar transform, the κ-value decreases but still shows “Moderate” to “Substantial” agreement among listeners. For patients with CP, the κ-values are lower compared to healthy subjects, but the results still suggest “Moderate” agreements among listeners.

**Table 4:** **Inter-rater agreement across 4 listeners**.

**Group**	**Muscle**	**Transform**	**κ**	Quality^*^
Healthy	BR	Cart.	0.810	Almost perfect
		polar	0.580	Moderate
	FPB	Cart.	0.817	Almost perfect
		polar	0.616	Substantial
CP	BR	Cart.	0.539	Moderate
		polar	0.459	Moderate

* *The quality of raters agreement is assessed according to Landis and Koch (1977)*.

## Discussion

We have shown that it is feasible to produce English vowels using myoelectric signals from non-speech muscles. When using Cartesian transformation between EMG and formant frequency, the fraction of correctly identified vowels is greater than 80% in healthy adults and 50% in two subjects with dystonia. The κ-test suggests that a naive listener is able to identify the vowel and the intelligibility is unlikely due to pure guessing. We have succeeded in extracting high-bandwidth, low variability control signals from non-speech muscles by using the non-linear Bayesian filtering algorithm. Our other main innovation is mapping the vowel generation to a virtual reaching movement on a 2D plane, which is intuitive even for children with movement disorders (see explanations below). With only 10 min of practice the subjects were able to produce intelligible English vowels.

We point out that the goal of this study is not to prove that myocontrolled speech synthesis can improve the intelligibility of speech in CP, but to test in this population whether it is feasible to produce the simplest human speech using flexible myocontrol provided by non-speech muscles. Our results first suggest that it is feasible to use upper-limb muscles to produce intelligible English vowels in real-time. Secondly, the success rate in CP (57% in Cartesian transform) after less than 20 min of familiarization is comparable with the reported speech intelligibility (~60%) in adult patients with CP (Platt et al., 1980), allowing us to further optimize the intelligibility by testing various EMG-formant mappings and muscle selections.

### Linkage to vocal music as innovative therapies

We plan on expanding our EMG-auditory paradigm to “virtual singing” for children with CP. Our EMG-formant mapping is one of its first kind to enable such goal. We suggest the use of assisted vocal music as a therapeutic approach to restore the social, emotional and cultural aspect of patients with CP, so to enhance their qualities of life (Flanagan, 1978). In this study we investigated the efficacy of EMG-formant mapping for controlling the intonation and tempo of speech. This major feature also naturally enables the “virtual singing.” To the audience of music training, we hope this seminal study can be of help and inspiration.

### Advantages and constraints due to speech-reaching mapping

One exciting discovery is that when using formant-based synthesis, the continuous production of vowels can be mapped to a continuous 2D reaching task. Similar ideas were presented in computer-aided education for pronunciation (Hiller et al., 1993), and commercialized software such as Vowel Viz (SmartPalate International, LLC, available for iOS devices). In these applications, the position of the cursor / finger was compared to pre-defined targets, and the best-fit vowel was chosen as the current sound. This category-selection approach did not allow modifications of sub-category features such as formant undershoot—features that healthy speakers actively control according to their desired speaking style (Hardcastle and Hewlett, 1999). Also the synthesized speech would be insensitive to fine details of limb reaching movement, such as the smoothness of trajectory, or the variability around the intended vowel, if using category selection. In particular, our results show that the synthesized speech transitioned from /i/ and /ɑ/ naturally and smoothly compared to human speech (Figure 6); such transition would not be controllable by the user if using category selection. Overall, our design highlights the value of continuous speech synthesis with controllable sub-vowel features, which may only be reproduced from a continuous limb movement such as reaching.

Due to the prevalence of reaching movements during day-to-day life, we argue that by associating vowel production with reaching it will make the task significantly easier to learn. Furthermore, myocontrolled speech synthesis creates a new paradigm that allows us to test human proprioception in the context of speech. For example, the role of proprioception in jaw muscles have been studied in previous studies (Ostry et al., 1997; Larson et al., 2000; Shiller et al., 2002), we can now ask more questions such as whether the proprioceptive feedback from the limb engages more, similar or less modulation compared to jaw. In future work we will use muscles directly involved in reaching movements, presumably from the same arm but from different joints. Such a multi-muscle control paradigm will require decoding the latent control signals from multi-channel EMGs, which has been an intriguing but challenging goal in myocontrol studies.

Our immediate next step is to test whether the quality of vowel generation can be improved through practice. In the current study, most patients with CP (3 out of 4) requested more practice after 10 min. They were given 5–10 extra min to familiarize with the task. The amount of practice required for fluent vowel generation is not the focus of the current study but remains an important topic for future studies. Furthermore, vowel generation may only improve within a narrow range of muscle contraction, because high muscle contraction may induce fatigue whereas low muscle contraction generally has significant variability. This, however, provides an important criterion for optimizing the mapping between EMG and formant frequency.

### Comparison with other technologies

Unlike in spinal cord injury or brainstem stroke, in CP there remains a connection between the brain and the spinal cord, which makes EMG a useful read-out of movement-related activities in motor cortex. When filtered with a non-linear Bayesian algorithm, we argue that electromyographic control (myocontrol) is more advantageous for motor restoration in this patient population compared with alternative paradigms such as brain computer interface (BCI), eye gaze control, buttons, or touchscreens. In particular, BCI is either invasive or low in bandwidth when using scalp electrodes. Eye gaze control is non-invasive but many children with CP have poor oculomotor control; also gaze interfaces restrict where the child can look. Buttons are low bandwidth and have limited ability for flexible tasks like speaking. Touchscreens require accurate reaching and multi-muscle control of the arm, which are often not possible for children with severe CP. Using formant-based synthesis we are able to achieve our standards for vowel production using only 2 independent EMG channels. We expect to be able to find 4–6 independent EMG channels in children with CP and explore the sufficiency to produce consonant-vowel syllables using up to 6 independent channels.

### Factors that may affect the performance

Our results showed that in both healthy subjects and patients with CP, Cartesian transform suits the vowel generation task better than polar transform. The subjects generally reported that Cartesian transform was more “intuitive” to control. This might be because that only one muscle from each limb was used in the current set-up. We predict that if we can access all the muscles involved in hand supination/pronation, the polar transform will be significantly easier to control. In addition, the thumb flexor (FPB) is better than brachioradialis for the task in healthy subjects, this might be due to that the precision of hand muscles is usually high among skeletal muscles.

In patients, the worst performer was a 12-year-old boy (patient #1 in Table 1) who had severe dysarthria, but it was noticed that he was easily distracted by the surroundings. Therefore, it is unclear whether the poor performance of patient #1 was due to inherent motor deficit or attention. The best performer was the 12-year-old girl who showed no less impairment in her diagnosis (patient #4 in Table 1). It has been well documented that significant muscle co-contractions are present in CP (Sanger et al., 2003), therefore the decreased performance of CP in this study could be due to the various co-contraction levels across patients, especially when the required muscle activation is high (sometimes > 60% MVC, Table 2). More experiments are needed to test what factors affect the performance in CP. It can be seen from Table 1 that the 4 patients showed a variety of motor deficits. It suggests that the feasibility of EMG-driven vowel synthesis is unlikely to be an anecdotal success specialized to only certain types of motor deficits.

### Consonant and syllable synthesis

Toward the goal of synthesizing intelligible English language, the most challenging step is perhaps creating consonants and eventually consonant-vowel syllables. Unlike vowels, the consonants are not only dominated by the steady-state frequency of F1 and F2, but the formants also must be produced with precise timing. Even more challenging is that, for some consonants, the entire consonant production occurs in only tens of milliseconds, meaning that millisecond-level control of formants may be required. In addition to the timing, several consonants will have to be synthesized with more formants than just F1 and F2 (Klatt, 1980) (e.g., F3 in /r/) and other types of acoustic output (e.g., frication in fricatives). Our design does not restrict the number of formants or types of acoustic outputs, but since adding more acoustic outputs to control will require additional EMG channels, this will increase the difficulty and the need for training for users. For comparison, even in normal children, the acquisition of intelligible speech production takes several years (Sander, 1972). In the current setup, the sound production is non-stop as there is no direct control of the volume. This makes it difficult to simulate stop consonants that require momentary break in the voice. Therefore, in consonant synthesis it will be necessary to map the volume (at least the on/off of voice) into movement space. Finally, we will need to test the overall speed of vowel and consonant production in order to determine the number of muscles needed, and whether synthesized speech can approach the speed of normal human communication. If we are successful, these techniques will produce a new technology for assistive communication that will allow children to communicate not only the declarative content of language, but also the individual social and emotional content that is so important for interacting with peers, teachers, and parents.

### Conflict of interest statement

The authors declare that the research was conducted in the absence of any commercial or financial relationships that could be construed as a potential conflict of interest.
